# Crawl positioning improves set-up precision and patient comfort in prone whole breast irradiation

**DOI:** 10.1038/s41598-020-72702-3

**Published:** 2020-10-02

**Authors:** Pieter Deseyne, Bruno Speleers, Wilfried De Neve, Bert Boute, Leen Paelinck, Vincent Vakaet, Hans Van Hulle, Max Schoepen, Michael Stouthandel, Annick Van Greveling, Giselle Post, Jan Detand, Chris Monten, Herman Depypere, Liv Veldeman

**Affiliations:** 1 Department of Radiation Oncology, Ghent University Hospital, C. Heymanslaan 10, 9000 Gent, Belgium; 2 Department of Human Structure and Repair, Faculty of Medicine and Health Sciences, Ghent University, C. Heymanslaan 10, 9000 Gent, Belgium; 3 Industrial Design Center, Faculty of Engineering and Architecture, Ghent University, Marksesteenweg 58, 8500 Kortrijk, Belgium; 4 Breast and Menopause Clinic, Ghent University Hospital, C. Heymanslaan 10, 9000 Gent, Belgium

**Keywords:** Breast cancer, Radiotherapy

## Abstract

Prone positioning for whole-breast irradiation (WBI) reduces dose to organs at risk, but reduces set-up speed, precision, and comfort. We aimed to improve these problems by placing patients in prone crawl position on a newly developed crawl couch (CrC). A group of 10 right-sided breast cancer patients requiring WBI were randomized in this cross-over trial, comparing the CrC to a standard prone breastboard (BB). Laterolateral (LL), craniocaudal (CC) and anterioposterior (AP) set-up errors were evaluated with cone beam CT. Comfort, preference and set-up time (SUT) were assessed. Forty left and right-sided breast cancer patients served as a validation group. For BB versus CrC, AP, LL and CC mean patient shifts were − 0.8 ± 2.8, 0.2 ± 11.7 and − 0.6 ± 4.4 versus − 0.2 ± 3.3, − 0.8 ± 2.5 and − 1.9 ± 5.7 mm. LL shift spread was reduced significantly. Nine out of 10 patients preferred the CrC. SUT did not differ significantly. The validation group had mean patient shifts of 1.7 ± 2.9 (AP), 0.2 ± 3.6 (LL) and − 0.2 ± 3.3 (CC) mm. Mean SUT in the validation group was 1 min longer (P < 0.05) than the comparative group. Median SUT was 3 min in all groups. The CrC improved precision and comfort compared to BB. Set-up errors compare favourably to other prone-WBI trials and rival supine positioning.

## Introduction

Breast cancer is the second most frequent cancer in women worldwide^1^. Early stage patients undergo breast conserving surgery. Additional whole-breast irradiation (WBI) leads to a 15.7% absolute reduction of relapse risk at 10 years and an absolute breast cancer mortality reduction of 3.3% at 15 years^2^. WBI implicates irradiation of surrounding organs at risk (OAR), which may cause side-effects like pneumonitis, fibrosis, ischemic heart disease, hypothyroidism, skin changes, and radiation-induced cancers^3–7^.

Intensity-modulated radiotherapy and volumetric-modulated arc therapy made radiation more conformal. Image guided radiotherapy (IGRT) made treatments more accurate and precise, decreasing OAR doses^8–10^. OAR doses are further improved by breath hold techniques^11,12^, a contralateral breast-holder, or changing the treatment position. Prone positioning minimizes target movement, reducing anterior thoracic expansion during breathing^13–15^. As the breast falls away from the thoracic wall, OARs can be more easily spared^16,17^. Prone positioning, however, is reported to suffer from reduced set-up precision, increased set-up time and reduced patient comfort^14,15,18^.

In case of regional nodal irradiation (RNI), prone breastboards often obstruct optimal beam paths. Their placement on top of the treatment couch hinders sagittal positioning of the laser on the target. Therefore, reference lines on the patient’s back are used, causing latero-lateral inaccuracies. This is aggravated by a tilted wedge in many breastboards, causing some patients to slide down from it. Furthermore, many patients have difficulties raising their arms above the head after surgery. We developed a support couch that addresses the problems of inaccuracy, discomfort and restricted beam access in classic prone breastboards. We named it the *prone crawl breast couch (further called crawl couch)*, because patients take a position resembling a phase of prone crawl swimming^19^. Dosimetric studies show reduced OAR doses with the crawl couch over supine setup in WBI + RNI^20,21^. However, there are no data available on set-up precision and comfort of the crawl couch.

In this study, we compare the crawl couch to our standard breastboard in a group of 10 patients refered for WBI, with regard to set-up precision, set-up time, and patient comfort. A second cohort of forty patients served as a validation group. The novelty of this trial is the use of the crawl couch, specifically designed to improve upon the limitations of “standard” prone positioning.

## Materials and methods

### Study design

Ten patients referred for hypofractionated WBI in 15 fractions were randomized 1:1 to start radiotherapy on either of the two available support devices. After 8 fractions, they crossed over the other device, serving as their own control. We further refer to this group as the *comparative group*. The trial was approved by the Ghent Universitary Hospital Ethics Board (reference number: EC-UZ-2014/1250, Belgian Registration Number: B670201422932). A second group of 40 patients was treated solely on the crawl couch and consented for their data to be used as internal validation of the results for the first patient group (reference number: EC-UZ-2016/0351, Belgian Registration Number: B670201628048). This group is further referred to as the *validation group*. All patients in our research voluntarily joined the study and informed consent was obtained from all participants before inclusion. The research was performed in accordance with relevant guidelines and regulations.

### Support devices

**Figure 1:**
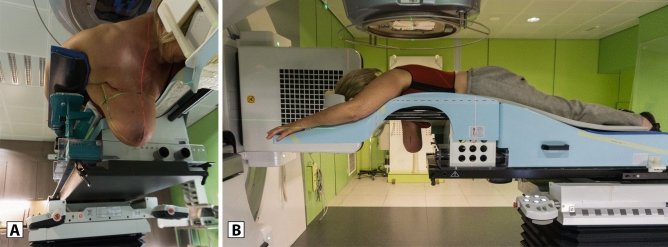
Patient set-up on a crawl couch prototype. (**A**) The prototype allows unobstructed anterior access to the ipsilateral lymphatic drainage areas. The projection of a red floor laser is seen along the sagital plane. (**B**) The contralateral side resembles current prone breastboards, overhanging design allows projection of a floor laser to decrease lateral positioning errors, while the patient is wearing a custom made unilateral bra, which retracts the contralateral breast away from the target volume.

Currently, in WBI-only treatments, we use a modified AIO prone breastboard (Orfit, Wijnegem, Belgium), described previously^22,23^ and further called *standard breastboard*. The *crawl couch* was designed for RNI in prone position. The contralateral arm is extended forward on an arm support, and the ipsilateral arm is held along the torso, leaving the ipsilateral shoulder and clavicular region unsupported by the couch. Stability is provided by the anhedral slope design of the contralateral arm support, an ipsilateral lateral pelvic support and an ipsilateral arm support blade along the patient’s waist and thoracic wall, which supports the arm up to the region below the axilla^24^. The caudal part of the *crawl couch*—supporting the patient’s legs hips and waist—rests on the treatment couch and the cranial part—supporting the torso breast and head—extends into the air, allowing sagittal projection of a floor laser on the treated breast^19^ (Fig. 1).

### Patient groups

Ten consecutive breast cancer patients referred for WBI were included in the comparative group. To avoid two extra CT-scans for breathhold planning (one on each treatment device), we allowed only right-sided WBI breast cancer patients for the control group. For the validation group of 40 patients, left- and right-sided breast cancers were included, as they were only treated using the *crawl couch*. For all patients, WBI was planned with a median prescription dose of 40.05 Gy in 15 fractions. Left-sided breast cancer patients were treated using a breath hold technique only if the planning constraints to the heart were not met (mean heart dose < 2 Gy and D02 < 10 Gy) in free breathing and were superior using the breath hold technique.

### Simulation

In the comparative group, patients underwent a CT-simulation on both support devices on the same day. Slice thickness was 5 mm. The contralateral breast was pulled laterally, by means of a unilateral bra [Tricolast, Deinze, Belgium]. A radio-opaque wire was placed around the ipsilateral breast. The isocenter was located on the acquired images and projected onto the patient using a laser co-ordinate system. The projected lines were marked on the patient’s skin with semi-permanent markers using different colours for each device. As the patient is lying on a solid blade couch, there is no floor laser projection available directly on the treated breast. This is an issue for the laterolateral positioning line, which is marked on the back of the patient. At the first treatment fraction, the patient is positioned using this laterolateral skin marking, and after positioning and CBCT correction, skin markings are added, using additional lasers. We use a floor laser for the crawl couch, and lasers at |40°| angles to the sagittal plane for the standard breastboard^22^. In this way, both devices had the same set-up protocol on our simulator. The validation group only underwent simulation using the crawl couch. All left-sided breast cancer patients underwent CT simulation including breathhold CT and free breathing CT. The free breathing CT was used for marking the isocenter in all patients.

### Endpoints

The primary endpoint was patient positioning accuracy & precision. Secondary endpoints were set-up time and patient comfort.

Positioning was assessed by daily CBCT after positioning on skin markings. Online matching of CBCT and simulation CT images allowed evaluation of position shifts in 3 axes: laterolateral (LL), craniocaudal (CC) and anteroposterior (AP). These were used to compare set-up accuracy & precision on the two support devices. Our treatment couches do not permit rotational corrections. Because additional set-up lines are marked on the patient at the time of the first fraction, couch shifts and set-up time at the first fraction of both devices were not used in the analysis.

We calculated systematic and random errors in accordance with van Herk^25^. The group systematic error, M, is defined as the mean of the mean individual patient shifts. Σ, defined as the standard deviation (SD) from M, is an estimator for the SD of group systematic errors. Finally, σ, an estimator of the SD of random error, is defined by the root mean square (RMS) of SD from the mean individual patient shift. We report these parameters and an estimated required planning target volume (PTV) margin using his formula, $2.5\sum + 0.7\sigma$ to compare our results to historical results.

Positioning time was registered in minutes by a radiotherapy technician (RTT) from the moment the patient climbed the treatment couch, until the CBCT was initiated. There was about 22% missing data because the treating RTT forgot to register either the start or stop time. Therefore, a Mann–Whitney U test was used to compare mean positioning time per device in the comparative group and also between the validation group and the comparative crawl group. We compared the means per patient per device using a paired Wilcoxon signed rank test in the comparative group.

Comfort and preference were assessed using patient reported outcome measures questionnaires (PROMs). Patients were asked 3 yes/no questions: if there were difficulties taking the treatment position, if they felt like they were sliding of the wedge, and if they felt tense while maintaining position. This was complemented with a visual analogue scale for pressure, tension, or pain sensation on multiple localisations (see supplementary information Figs. 1 and 2). PROMs were filled in at simulation and after each of the 2 treatment parts. After completing treatment, patients were asked which device they preferred. The validation group reported their PROMs after simulation, day 5 of treatment and at the end of treatment.

Data were analysed using R 3.3.2 with paired comparisons where possible. Tests were performed 2-sided with an α-value of < 0.05. One patient did not return a questionnaire form after the first treatment part, and one patient failed to answer the yes/no question asking about tension. In both cases the questions pertained to the crawl couch and were categorized as missing.

## Results

### Set-up accuracy & precision

**Figure 2:**
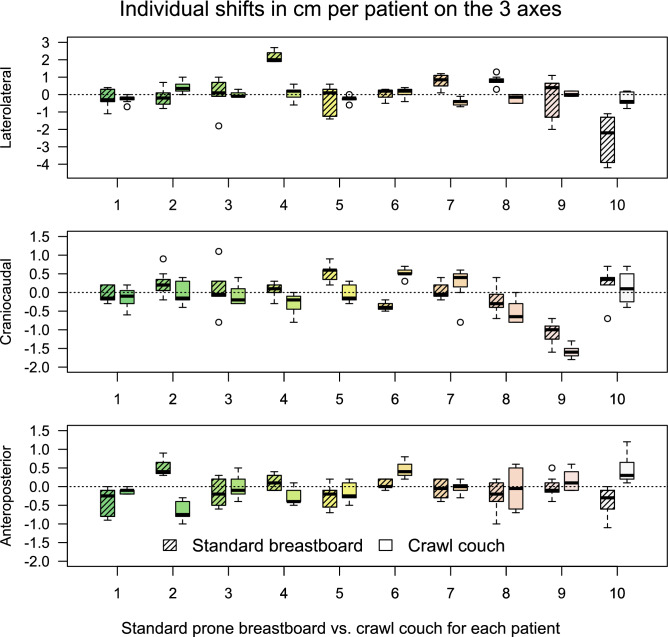
Distribution of individual patient shifts registered on 3 axes for the standard and crawl prone positioning devices. Shifts were extracted by positioning patients on reference lines and noting the shift needed to match the CBCT to the simulation CT as closely as possible. Each patient is represented by a separate colour.

Table 1 shows descriptive statistics for the comparative group on the 3 axes. Figure 2 shows the shifts along the 3 axes acquired during the treatment per patient in this group. A paired T-test was performed to compare M for positioning data along the same axis on both support devices, and an F-test was performed for difference between variances of patient means for the 3 axes on both support devices. There was no significant difference between mean shifts on the 2 support devices, but the SD was significantly smaller for the LL axis for the crawl couch compared to the standard breastboard (P < 0.001). No significant differences in SD for the other axes were observed. The RMS as an estimator for SD of random error is based on individual patient SDs. The individual patient SDs, when treated as a separate measurement, had a statistically significant different mean (P < 0.05) and SD (P < 0.001) along the LL axis only, with smaller values for the crawl couch compared to the standard breastboard.

**Table 1:** Means ± standard deviations per patient of shifts in direction of 3 axes on standard breastboard vs crawl couch (comparative group) (in mm).

Patient	AP		LL		CC	
	Standard	Crawl	Standard	Crawl	Standard	Crawl
1	− 3.8 ± 3.8	− 1.3 ± 0.8	− 2.3 ± 5.4	− 2.6 ± 2.3	− 0.7 ± 2.2	− 1.4 ± 2.8
2	5.1 ± 2.3	− 6.7 ± 2.7	− 1.7 ± 5.5	4.2 ± 3.5	2.4 ± 3.6	− 0.3 ± 3.1
3	− 1.7 ± 3.7	0 ± 3.2	0 ± 9.8	0.1 ± 1.7	0.7 ± 6.2	− 0.7 ± 2.8
4	1.2 ± 2.0	− 2.6 ± 2.5	21.5 ± 3.3	0.6 ± 3.9	0.7 ± 2.1	− 3 ± 2.8
5	− 2.9 ± 3.2	− 1.7 ± 2.7	− 3.7 ± 8.9	− 2.5 ± 2	5.1 ± 2.3	− 0.5 ± 2.4
6	0.7 ± 1.3	4.5 ± 2.3	0.3 ± 3.1	1.2 ± 2.9	− 3.7 ± 1.3	5.2 ± 1.3
7	− 0.7 ± 2.5	− 0.3 ± 1.7	7.7 ± 4.1	− 4.4 ± 2.1	0.3 ± 2.3	2.1 ± 4.9
8	− 2.3 ± 4.2	− 0.5 ± 5.8	8 ± 3.1	− 2.2 ± 2.3	− 2.1 ± 3.6	− 5.3 ± 3.2
9	− 0.1 ± 2.9	1.7 ± 2.9	− 2.6 ± 12.5	0.3 ± 1.4	− 10.9 ± 3.1	− 15.8 ± 1.8
10	− 4.0 ± 4.0	4.7 ± 4	− 24.8 ± 13.2	− 2.4 ± 4	2.2 ± 4.8	1.3 ± 4.4
Group systematic error (M)	− 0.8	− 0.2	0.2	− 0.8	− 0.6	− 1.9
Group systematic error SD (Σ)	2.8	3.3	11.7*	2.5*	4.4	5.7
Root mean square of SD’s (σ)	3.1	3.1	7.8*	2.8*	3.4	3.1
Required margins (mm)	9	10	35	8	13	16

AP = anteroposterior shift, LL = laterolateral shift, CC = craniocaudal shift, SD = standard deviation, *P < 0.05.

Shifts along each axis for all crawl groups are reported in Table 2 while Table 3 shows the data for each patient and shows the subgroups for left versus right-sided breast cancer. The sign of the LL shift was inverted in left-sided patients, mirroring them to match the right-sided patients. When comparing crawl comparative and crawl validation positioning using an unpaired T-test, there was a significant difference in M for the AP shifts (p < 0.001), but not for CC and LL directions. F-test was significant for the LL and CC axis, but not for the AP axis. Individual patient SDs, treated as a separate measurement, had a statistically significant different mean (P < 0.01) along the LL axis only, with larger values for the validation group, but no significant difference in SD per axis between study groups.

**Table 2:** Van Herk’s parameters for crawl couch margin calculation in the comparative versus the validation group (in mm).

Patient	AP			LL			CC		
	Comparative	Validation	All	Comparative	Validation	All	Comparative	Validation	All
Group systematic error (M)	− 0.2	1.7	1.4*	− 0.8	− 0.3	− 0.5	− 1.9	− 0.2	− 0.5
Group systematic error SD (Σ)	3.3	2.9	3.1	2.5	3.6	3.4*	5.7	3.3	3.9*
Root mean square of SD’s (σ)	3.1	3.5	3.4	2.8	4.5	4.2*	3.1	3.5	3.4
Required margins (mm)	11	10	10	8	12	11	16	11	12

AP = anteroposterior shift, LL = laterolateral shift, CC = craniocaudal shift, SD = standard deviation, *significant difference between comparative and validation group (p < 0.05).

**Table 3:** Means and standard deviations per patient of shifts in direction of 3 axes on crawl couch (validation group) by breast cancer laterality (in mm).

Patient	Left			Patient	Right		
	AP	LL	CC		AP	LL	CC
1	− 1.4 ± 2.6	6.7 ± 4.9	− 2.1 ± 2.6	21	1.2 ± 2.5	− 2.9 ± 3.2	− 1.6 ± 3.3
2	5.9 ± 2.2	4.1 ± 2.5	2.3 ± 2.3	22	0.5 ± 2.1	− 3.5 ± 3.5	0.4 ± 2.4
3	1.7 ± 2.9	− 3.6 ± 4.5	0.9 ± 3.3	23	0.9 ± 2.9	1.4 ± 5.4	− 3.1 ± 3.4
4	− 0.3 ± 2.3	0.4 ± 2.9	− 1.1 ± 2.3	24	− 0.5 ± 1.7	− 1.8 ± 3.3	− 5.5 ± 3.2
5	4.9 ± 4.5	− 1.9 ± 7.9	4.3 ± 4.8	25	0.8 ± 2.9	− 1.6 ± 2.2	− 5.9 ± 4.7
6	2.4 ± 3.3	− 0.4 ± 2.5	2.4 ± 2.8	26	1.3 ± 2.4	− 3.9 ± 4.6	− 6.4 ± 4.3
7	4.5 ± 3.8	− 3.7 ± 3.2	− 1.4 ± 2.9	27	3.3 ± 2.0	2.8 ± 3.8	− 3.5 ± 3.5
8	4.5 ± 4.2	9.5 ± 6.0	3.3 ± 1.8	28	7.4 ± 2.2	2.4 ± 3.9	− 6.5 ± 4.2
9	− 1.1 ± 1.7	6.3 ± 6.2	5.0 ± 2.8	29	− 4.2 ± 4.4	0.8 ± 3.4	− 6.2 ± 3.4
10	2.4 ± 2.5	6.8 ± 3.4	3.9 ± 2.7	30	9.5 ± 8.7	3.3 ± 10.1	0.1 ± 4.7
11	− 1.1 ± 2.1	− 10.6 ± 2.6	− 0.7 ± 2.1	31	4.3 ± 5.3	− 1.1 ± 4.8	− 0.2 ± 2.9
12	1.9 ± 2.9	− 1.5 ± 3.7	1.3 ± 2.6	32	2.4 ± 2.6	0.0 ± 3.4	3.9 ± 3.8
13	1.4 ± 2.7	3.4 ± 9.2	0.0 ± 4.3	33	3.7 ± 6.6	1.9 ± 4.1	2.1 ± 5.1
14	6.2 ± 3.4	0.5 ± 4.0	0.1 ± 4.4	34	5.1 ± 2.7	− 0.2 ± 5.6	2.4 ± 5.2
15	1.1 ± 3.4	− 0.1 ± 4.8	4.9 ± 4.3	35	− 1.4 ± 5.7	1.1 ± 3.5	− 3.9 ± 4.0
16	− 0.9 ± 3.1	− 2.7 ± 4.4	− 1.0 ± 3.7	36	− 1.1 ± 2.7	0.4 ± 2.4	0.1 ± 3.5
17	− 1.2 ± 3.1	− 0.8 ± 3.1	3.2 ± 4.2	37	1.7 ± 1.9	0.8 ± 3.2	− 5.3 ± 1.9
18	3.8 ± 1.8	− 3.1 ± 3.3	0.2 ± 2.0	38	3.6 ± 5.4	− 2.5 ± 2.6	− 1.0 ± 2.4
19	− 2.2 ± 2.9	0.4 ± 4.0	3.4 ± 3.2	39	0.3 ± 2.5	− 3.4 ± 3.0	− 1.7 ± 2.9
20	1.8 ± 1.1	3.1 ± 3.1	1.9 ± 2.6	40	− 2.6 ± 4.0	0.0 ± 5.4	3.1 ± 3.9
Group systematic error (M)	1.7	0.6	1.5		1.8	− 0.3	− 1.9
Group systematic error SD (Σ)	2.6	4.7	2.2		3.3	2.2	3.4
Root mean square of SD’s (σ)	2.9	4.6	3.2		4.0	4.4	3.7
Required margins (cm)	9	15	8		11	9	11

AP = anteroposterior shift, LL = laterolateral shift, CC = craniocaudal shift, SD = standard deviation.

### Time registration

Table 4 shows measurement data for both treatment devices. In the comparative group, there was no significant difference in set-up time between the two support types (P > 0.717) using the unpaired Mann–Whitney U test. The paired Wilcoxon signed rank test on the means per patient also showed no significant difference in set-up time between the two devices (P > 0.952). The mean positioning time on the crawl couch was significantly longer for the validation group compared to the comparative group (P < 0.001). However, the median positioning time is the same between all groups (3 min).

**Table 4:** Descriptive statistics showing treatment set-up duration for standard and crawl prone support device.

	Valid	Missing	Mean (minutes)	Standard deviation	Median (minutes)	Maximum (minutes)	Missing data (%)
Standard prone breastboard	46	19	2.7	0.92	3	5	29
Prone crawl couch	55	10	2.8	0.98	3	5	15
Validation group prone crawl couch	443	117	3.8	1.50	3	19	21

### Pressure/tension points and pain

**Figure 3:**
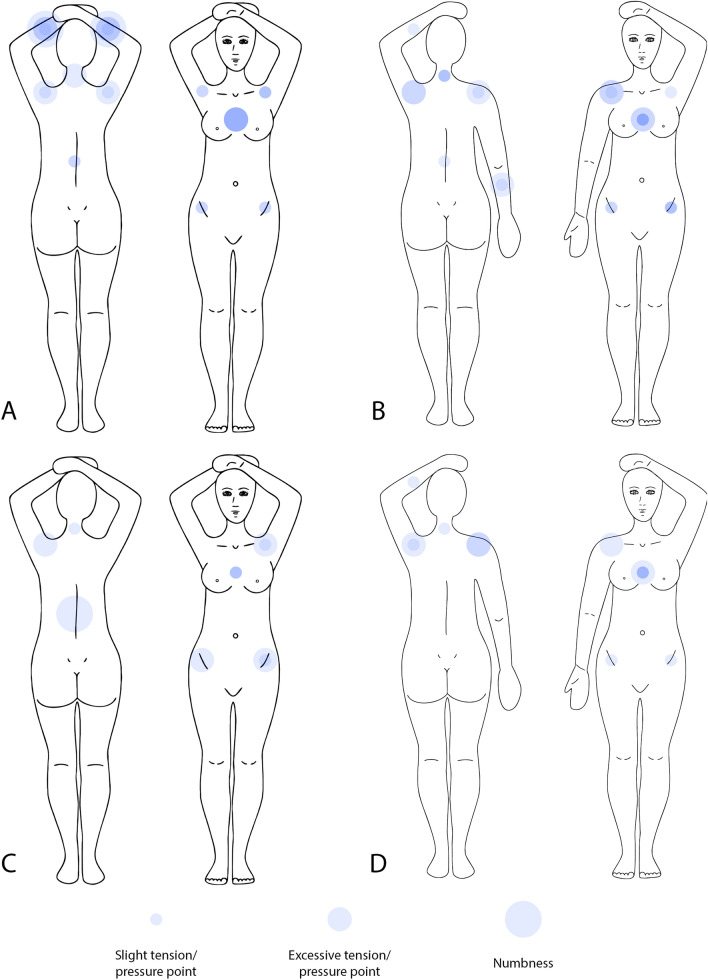
Pressure/tension scores for the 11 different localizations as indicated by individual patients in the comparative group, to be viewed side by side with Fig. 4. Different circle sizes indicated different pressure/tension scores per patient. Overlapping circles intensify the circle colour. (**A**) Standard prone breastboard at simulation, (**B**) crawl couch at simulation, (**C**) standard prone breastboard after treatment (**D**) crawl couch after treatment.

**Figure 4:**
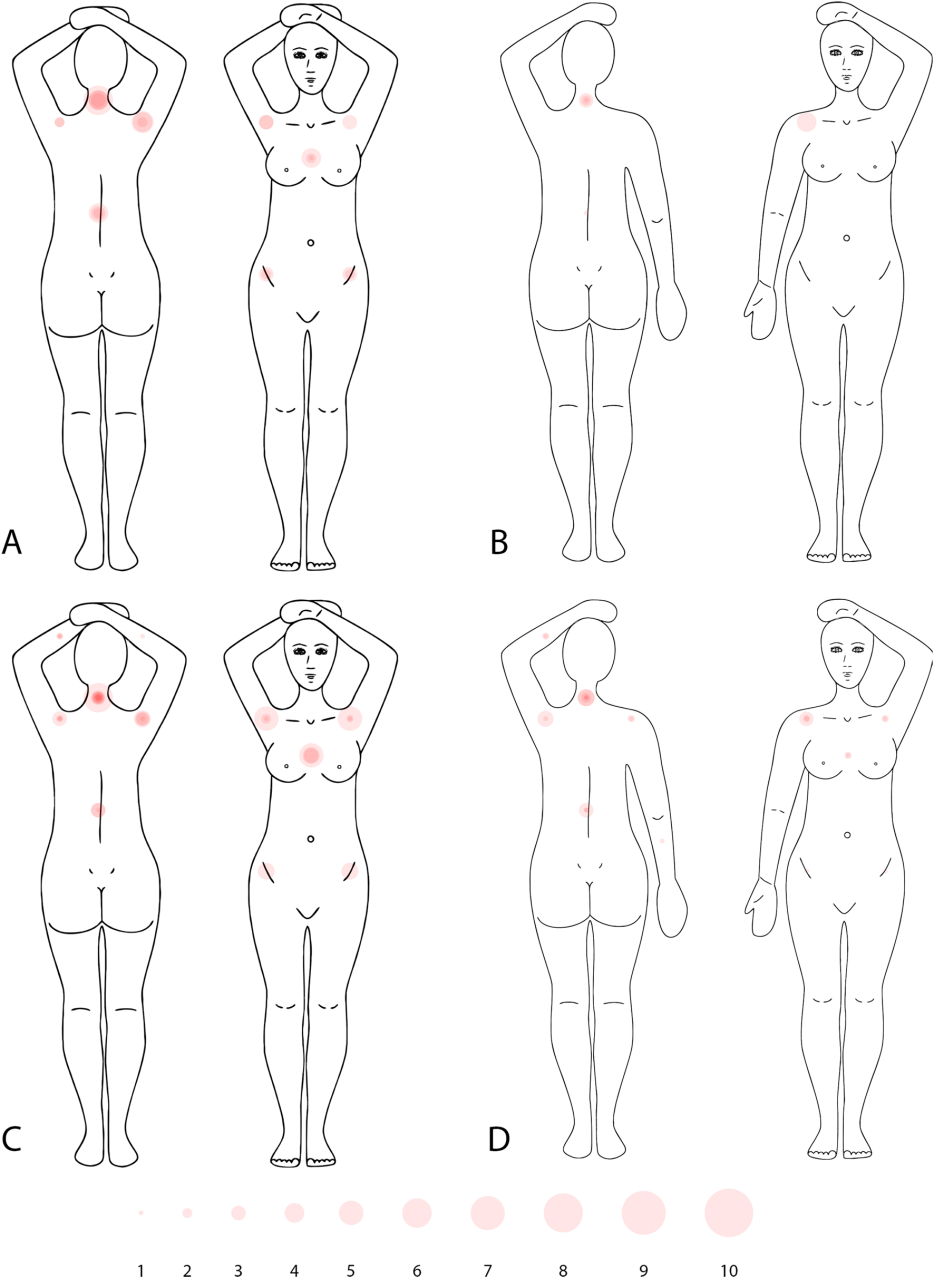
Pain scores for the 11 different localizations as indicated by individual patients in the comparative group, to be viewed side by side with Fig. 3. Different circle sizes indicated different pain scores per patient. Overlapping circles intensify the circle colour. (**A**) Standard prone breastboard at simulation, (**B**) crawl couch at simulation, (**C**) standard prone breastboard after treatment, (**D**) crawl couch after treatment.

**Figure 5:**
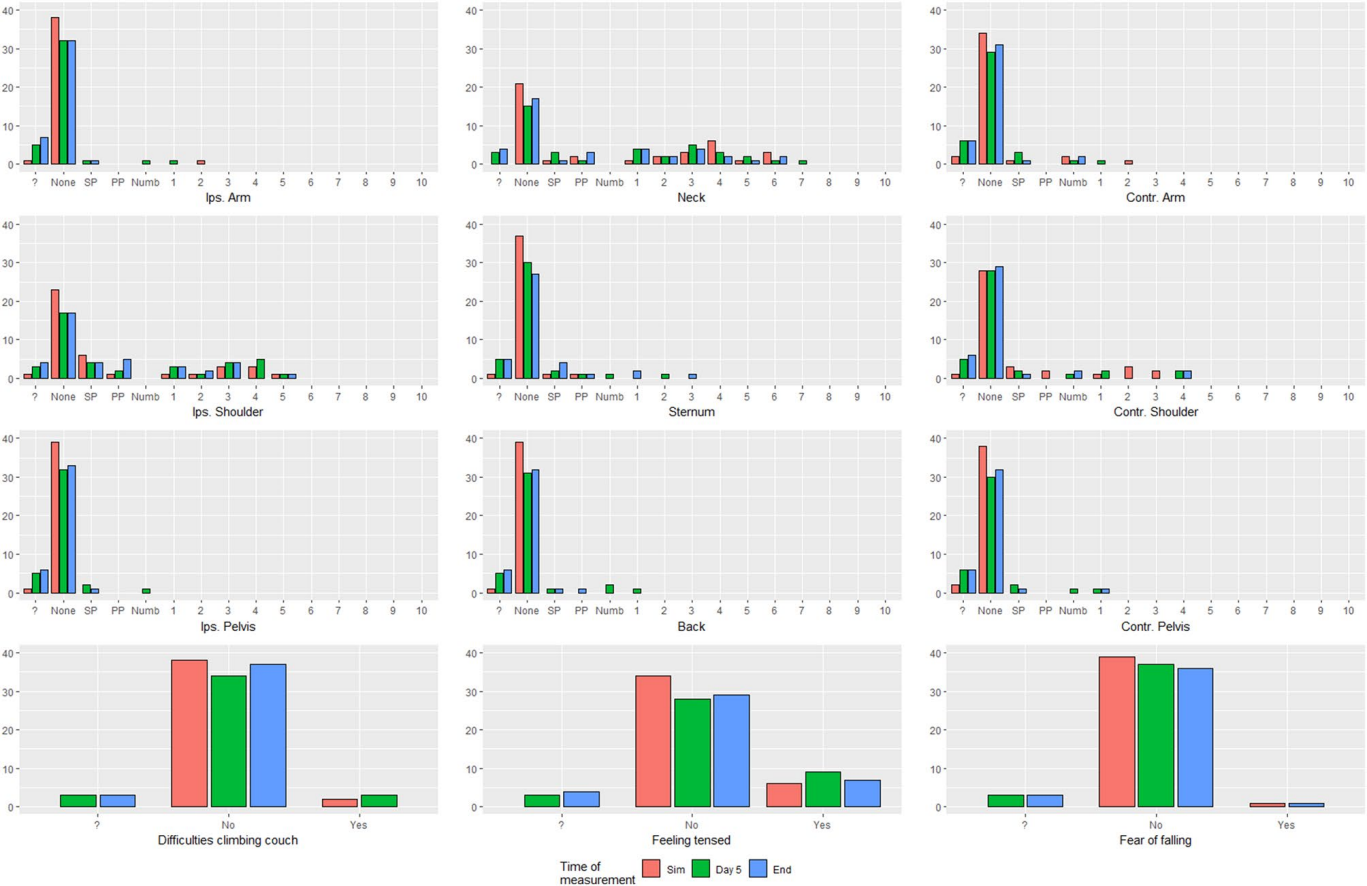
Scores on the pain/pressure scale and answers to yes/no questions for the validation group at simulation (end), on day 5 of treatment and end of treatment. ? = missing, SP = slight pressure, PP = pronounced pressure, Numb = numbness, Ips = ipsilateral, Contr = contralateral.

We asked to evaluate pressure/tension and pain on several anatomical points, scored on the continuous visual-analogue scale provided in supplementary information Figs. 1 and 2. This scale was separated in pressure/tension scores (Fig. 3) and pain scores (Fig. 4). This division was made because, while some pressure/tension is acceptable, we mainly aimed to visualise the effect of using either support device on pain sensation. This means that these figures can only be correctly interpreted side by side: a decreased score on Fig. 4 (pain) can result in an increased score on Fig. 3 (pressure/tension), because these data originate from the same visual-analogue scale. Figure 5 shows the scores for the validation group at each measurement point.

### Comfort and preference

A crosstable showing the response for the comparative to the yes/no questions per device is shown in Table 5. At simulation, only one patient felt tense on the crawl couch, and she experienced the same on the standard breastboard. After treatment, none of the patients who answered the questions and returned the form experienced any pain or pressure, sliding sensation or tension on the crawl couch, whereas, on the standard breastboard, this was the case for some patients. When asked to indicate which support device they preferred, 9 out of 10 patients preferred the crawl couch. Figure 5 shows the responses to the yes/no questions for the validation group at each measurement point.

**Table 5:** Crosstable showing yes/no questions for standard breastboard vs crawl couch in complete cases.

Simulation				After treatment			
Tension (n = 10)		Standard breastboard		Tension (n = 8)*		Standard breastboard	
		No	Yes			No	Yes
Crawl couch	No	5	4	Crawl couch	No	3	5
	Yes	0	1		Yes	0	0

Sliding sensation (n = 10)		Standard breastboard		Sliding sensation (n = 9)**		Standard breastboard	
		No	Yes			No	Yes
Crawl couch	No	8	2	Crawl couch	No	8	1
	Yes	0	0		Yes	0	0

Difficulties assuming treatment position (n = 10)		Standard breastboard		Difficulties assuming treatment position (n = 9)**		Standard breastboard	
		No	Yes			No	Yes
Crawl couch	No	7	3	Crawl couch	No	7	2
	Yes	0	0		Yes	0	0

n = Number of cases that returned the forms and responded to the question.

*1 patient did not respond and 1 patient did not return the response form, **1 patient did not return the response form.

## Discussion

This study was performed to evaluate the impact of the crawl couch on positioning accuracy & precision, timing and patient comfort in breast irradiation. First of all we discuss the comparative study group. As to accuracy of treatment, the difference in mean shifts (M) between both groups was not significant for any axis, but the crawl couch improved LL positioning, in terms of inter-patient (∑) as well as intra-patient variability (σ), improving precision.

This improvement in LL precision comes as a result of the design of the crawl couch: the armrest prevents the patient from sliding down the wedge, and the open design allows sagittal laser beam projection on the target tissue. This allows for smaller PTV margins, reduction of the dose to OARs, and it reduces the need for daily CBCT to evaluate random errors.

Image guided radiotherapy (IGRT) allows daily imaging, with possibilities for gating or tracking. These improvements in treatment techniques allow more accurate visualisation of the target, while treatment machines are increasingly capable of high precision and real-time treatment adaptation. Therefore, the continued use of Van Herk’s formula^25^ to calculate clinical PTV margins is becoming an anachronism. It is our opinion that in the era of IGRT, Van Herk’s formula and its parameters will mainly be used to compare positioning results to historical data, which we will do below.

We used van Herk’s parameters and formula to estimate the required margins along the 3 axes (Tables 1, 2, 3). We estimated a suggested anisotropic PTV margin for the whole group (50 patients) of 1.0, 1.1 and 1.2 cm for the AP, LL and CC axes, respectively. Our results are not always directly comparable to other published data, because margin recipes differ, or the recipe is not mentioned.

First, we compare our results to other reported results for prone WBI positioning. We updated the overview as cited by Mulliez et al.^18^ (Table 6), and calculated the margins according to the ($2.5\sum + 0.7\sigma )$ formula where possible, so as to make direct comparison as valid as possible. Comparing our margins to these recalculated margins, we see similar results as Mulliez et al.^18^, except for the LL margin, which was substantially reduced using the crawl couch. The standard prone position did show a larger LL error in our sample. Only the group of Varga^26^ reported vastly superior margin results, but their setup was evaluated with EPID using a single treatment beam as portal. There is an inherent loss of information when only using 2D imaging as verification, and indeed they do not report the errors along each axis separately. Data cited in the publication of Mitchell et al.^27^ back this up. They state in their manuscript that CBCT catches more errors than the EPID technique and thus will require larger margins. While Joszef et al.^28^ report slightly superior margins along the LL and CC axis compared to the prone crawl setup, their report only concerns partial breast irradiation, so effects of edema on patient shift might not be as pronounced. In general, our crawl couch results compare favourably to most of the results published for prone radiotherapy^13–15,18,22,26–30^, especially for the AP and LL systematic error distribution defined by M and ∑. Because our random error σ is lower than in these other studies, we can obtain smaller PTV margins of around 1 cm in these directions. However, despite our CC margins being comparable to other studies, they range amongst the wider margins reported. This seems to be caused by a higher ∑ rather than σ, as the latter is among the lowest observed in prone position, but the former is amongst the highest. This higher ∑ could be due to less reproducible shoulder support on the irradiated side, as this position has more possible variation using this new positioning technique. As σ is low, this larger ∑ has more to do with inter-patient rather than intra-patient variation. Because each individual patient’s position will be less variable between fractions, margins could be safely reduced using eNAL-protocols^31^.

**Table 6:** Setup errors in various prone and supine positioning setups.

Position	Author	Year	WBI/PBI	Pts	Method	Systematic error M (mm)			St. Dev systematic error ∑ (mm)			Random error σ (mm)			Margin (mm)		
						VE	LA	LO	VE	LA	LO	VE	LA	LO	VE	LA	LO
Supine	Ahunbay*^29^	2012	PBI	3	CT	–			–			–			13.9		
	Cai^32^	2010	PBI	10	CBCT	–			2.3	2.1	3.1	2.0	1.8	2.3	7.2	6.5	9.4
	Donovan^33^	2012	WBI	38	CBCT	–			1.2	1.5	1.2	2.9	3.0	3.2	5.0	5.9	5.2
	Hasan**^34^	2011	PBI	16	CBCT	9.25			–			–			10	10	10
	Kirby***^14^	2011	WBI	25	CBCT	3.4	1.6	0.1	1.8	1.8	1.9	4.2	2.7	3.1	9.6	9.7	10.2
									Without respiratory movement this yields other result:						7.4	6.4	6.9
	Penninkhof ^§^^35^	2012	WBI	80	EPID	–			2.8	2.3	2.4	2.3	2.1	2.0	8.6	7.2	7.4
	Shah ^§§^^36^	2013	WBI	50	AlignRT	0.1	− 0.2	− 0.6	2.6	1.2	1.4	3.2	2.2	2.2	8.6	4.5	5.0
	Topolnjak ^§§§^^37^	2011	WBI	21	CBCT	− 0.1	0.5	− 0.4	0.7	1.2	1.3	0.9	1.0	1.2	2.4	3.7	4.1
	van Mourik^38^	2011	WBI	19	CBCT	− 1.4	0.3	− 0.2	1.5	1.3	1.4	3.8	3	2.9	6.4	5.4	5.5
	Varga^26^	2009	WBI	61	EPID	–			0.8			3.0			4.1		
	White^39^	2007	PBI	20	CBCT	–			1.7	2.7	2.4	2.2	2.4	2.9	5.8	8.4	8.0
	Veldeman ‡^15^	2012	WBI	10	CBCT	2.8	− 1.5	1.4	5.1	4.5	2.4	3.2	7.3	2.3	15.1	16.4	7.5
	Mulliez^18^	2016	WBI	103	CBCT	–			3.1	2.7	2.8	3.8	3.6	3.4	10.4	9.4	9.4
	Cravo Sa^40^	2018	WBI	10	AlignRT	–			2.9	2.9	3.3	2.9	2.4	3.1	9.3	8.9	10.4
Prone	Ahunbay*^29^	2012	PBI	10	CT	–			–			–			13.9		
	Jozsef^28^	2011	PBI	70	CBCT	− 1.9	− 0.2	− 0.2	3.8	2.8	2.1	4.3	3.7	2.9	12.6	9.6	7.3
	Kirby***^14^	2011	WBI	25	CBCT	2.8	0.4	3.6	3.4	3.1	4.3	4.2	3.8	5.4	12.5	11.5	15.6
									Without respiratory movement this yields other result:						11.4	10.4	14.5
	Mitchell^27^	2010	PBI	10	EPID-surface	− 0.1	–	–	4.7	–	–	13.2	–	–	21.0	–	–
					EPID-fiducial	0.8	–	− 0.4	2.7	–	1.7	9.0	–	3.8	13.1	–	6.9
					Resp. motion	–			–			–			14.0	14.0	6.8
	Morrow^13^	2007	PBI	15	EPID	–			–			–			16	–	20
	Varga^26^	2009	WBI	61	EPID	–			0.9			3.9			4.9		
	Veldeman^22^	2010	WBI	10	CBCT	− 5.0	0.8	− 1.3	4.8	3.6	5.6	3.2	4.2	2.6	14.2	11.9	15.8
	Veldeman^15^	2012	WBI	10	CBCT	7.2	0.6	− 0.8	3.7	5.7	2.4	4.3	6.9	3.8	11.4	19.1	8.6
	Mulliez^18^	2016	WBI	139	CBCT	–			3.3	6.4	4.3	3.4	9.2	4.3	10.5	22.4	13.7
	Lakosi^30^	2016	WBI	36	CBCT	–			3.3	4.5	3.9	2.8	5.4	3.8	10.3	15.0	12.3
	Deseyne	Current	WBI	10	CBCT	− 0.8	0.2	− 0.6	2.8	11.7	4.4	3.1	7.8	3.4	9.2	34.7	13.4
Prone crawl	Deseyne	Current	WBI	50	CBCT	1.4	− 0.5	− 0.5	3.1	3.4	3.9	3.4	4.2	3.4	10.1	11.4	12.1

*Authors used other formula: $0.7\sum + 2 \sigma$. No direction of margins is specified.

**Systematic error calculated from magnitude of mean set-up error per patient. No directions are specified, it is unclear how the published margins were calculated.

***Reported margins include respiratory movement. Van Herk formula $2.5\sum + 0.7\sigma$ yields different results.

^§^Reported data from boost area using eNAL protocol.

^§§^Authors used other formula: $2\sum + 0.7\sigma$.

^§§§^Authors used other formula: $2\sum + 0.3\sigma$.

^‡^M is reported instead of ∑, but ∑ can be extracted from standard deviation of M, because individual mean shifts are provided.

Comparing the validation study group with the crawl comparative group, there was a difference in mean AP shift that was larger in the validation group, but there was no difference between the groups for LL or CC mean shift. This difference in M does not factor into Van Herk’s equation. The most pronounced changes seen for the crawl position in the comparative group, being the LL and CC shifts, were somewhat toned down in the comparison with the validation group. Due to a significantly larger Σ and σ along the LL axis in the validation group, the LL margin became larger, and a significantly smaller ∑ along CC axis in the validation group made the CC margin smaller.

The reason for the decreased CC shift in the validation group might be the learning curve, resulting in more reproducible shoulder positioning between patients. It is unclear why the LL shifts on the crawl couch are larger in the validation group than in the comparative group. Looking at breast cancer laterality in the validation group shows a large difference in LL shifts between left and right-sided breast cancers, to the detriment of the left-sided patients, while right-sided patients confirm the observed data from the comparative group. Again, the difference between the two groups is found in a larger ∑ in the left-sided group, while σ remains comparable between the groups. A possible explanation may be found in the number of simulation CTs.

At simulation, left-sided breast cancer patients received multiple scans (breathhold followed by free breathing) without changing their position between scans. Isocenter lines were then marked based on the free breathing scan, because the breast is assumed not to change position between breathhold and free breathing^12^. It is possible that the patient shifts position as a result of longer simulation time and multiple table movements during this period. This could result in a discrepancy between imaging and skin demarcation, possibly leading to larger shifts on the treatment device. This also explains why there is no directional preference for these laterolateral shifts between patients (i.e. no large group mean error). In contrast, breathhold does not explain the shifts, as positioning correction is based on a free breathing CBCT which is co-registered with the free breathing simulation CT. Preparation and positioning is therefore the same for breathhold and free breathing patients.

Next, we compare our set-up results for the crawl couch to the results for supine positioning^14,15,18,26,29,32–40^ (Table 6). We found our LL margins were higher than the mean of reported margins in these publications (8.1 mm). However, our margins are still lower than some of the reported margins in supine position. Furthermore, our ∑ is larger than most of the supine series, meaning that there is a higher grade of systematic error. The use of eNAL procedures with adequate image guidance should substantially decrease these errors and therefore the required margins. The AP margin for the crawl couch was higher than the mean margin (8.2 mm) in supine position, but lower than the highest registered supine margin (15.1 mm). The crawl couch’s CC margins are slightly larger than the margins in supine position (12.1 mm using the crawl couch, and 10.4 mm at most in supine position). These results show that our results of prone crawl positioning fall within the range of reported AP and LL margins for supine positioning, with only slightly larger CC margin requirements.

Although van Herk^25^ specifies that ∑ is actually the standard deviation from M, many authors report ∑ instead of M as systematic error, because it is used in the formula. However, disregarding M is wrong. While M remains small along most axes in the literature overview, the AP axis shows the biggest spread (− 5 mm up to 7.2 mm). This may lead to inaccurate interpretation of the calculated margins. Taking data from Veldeman et al.^15^ as an example, ∑ = 3.7 mm and σ = 4.3 mm for AP set-up error yields calculated PTV margins of 11.4 mm using van Herk’s formula. But, given M = 7.2 mm, this means that the mean patient shift will fall outside the margins calculated for M = 0 mm for about 13% of the population. Except for Veldeman et al.^15^, all authors in the overview included left and right-sided tumours, so systematic LL shifts would be corrected by different tumour laterality, resulting in M close to zero. Our study also corrected the sign of the LL shifts for left-sided breast cancer patients so as to not confound the effect on M.

We found no significant difference in set-up time between both support devices in the comparative group. Comparison with a previous report from our center by Veldeman et al.^15^ shows a shorter time needed for positioning for both support devices in the current study. This shows that a learning curve for implementation of prone positioning exists. However, the validation group had a mean positioning time that was 1 min longer than the comparative group on the crawl couch (3.8 vs. 2.8 min). Because about 22% timing measurements were missing, and the time was noted in minutes by different RTTs, we feel future time registration studies could benefit from automation. But although time differences of less than a minute cannot be detected in this study, the relevance of such differences can be questioned.

There was a near unanimous preference for the crawl couch. Regarding patient comfort, the crawl couch never performed worse than the standard breastboard. When evaluating pain and pressure/tension scores, we showed a substantial difference in favour of the crawl couch for the sternum at the time of simulation. The difference is probably most apparent at that time, because patients spend a longer time on the support device. Figures 3 and 4 illustrate the observed scores for all patients, and allow visual evaluation of the data. While the sample size might not allow to detect any differences perceived by these patients, it is quite clear from the graphs that the crawl couch causes the patient less pain.

While this trial reports on inter-fraction positioning variability on the crawl couch, we did not acquire data allowing us to evaluate the effect of intra-fraction motion and its impact on patient dosimetry. We assumed the intra-fraction motion to be negligible because there is evidence that the breast has no substantial respiration-related movement in prone positioning^13–15^. However, other factors influencing intra-fraction variation and their impact on dosimetry may merit further investigation.

## Conclusion

Compared to standard prone positioning, the use of the novel crawl couch improves the positioning precision for prone WBI. The crawl position can almost match set-up errors that are seen in supine positioning without large increases in additional treatment time. In addition, patients generally preferred the crawl couch over a standard prone breastboard and experienced less discomfort, showing lower pain and pressure scores on the crawl couch.

## Supplementary information

Supplementary Figures.

## Data Availability

Data analysed during this study are available from the corresponding author on reasonable request within the confines of EU General Data Protection Regulations.
