# Supplementary material for: Crawl positioning improves set-up precision and patient comfort in prone whole breast irradiation
Source: Sci Rep. 2020 Oct 2;10:16376. doi: 10.1038/s41598-020-72702-3 (PMC7532156; doi:10.1038/s41598-020-72702-3)

# Supplementary information:

**Title:** Crawl positioning improves set-up precision and patient comfort in prone whole breast irradiation

**Author information:** Deseyne Pieter, MD^1,2,^*; Speleers Bruno^2^; De Neve Wilfried, MD, PhD^1,2^; Boute Bert, MSc, PhD ^2,3^; Paelinck Leen, PhD^1^; Vakaet Vincent, MD^1,2^; Van Hulle Hans, MA²; Schoepen Max, MSc³; Stouthandel Michael, MSc²; Van Greveling Annick^1^; Post Giselle^2^; Detand Jan, MSc, PhD^3^; Monten Chris, MD, PhD^1,2^; Depypere Herman, MD, PhD^2,4^; Veldeman Liv, MD, PhD^1,2^;

^1^ Department of Radiation Oncology, Ghent University Hospital, C. Heymanslaan 10, B-9000 Gent, Belgium

^2^ Department of Human Structure and Repair, Faculty of Medicine and Health Sciences, Ghent University, C. Heymanslaan 10, B-9000 Gent, Belgium

^3^ Industrial Design Center, Faculty of Engineering and Architecture, Ghent University, Marksesteenweg 58, B-8500 Kortrijk, Belgium

^4^ Breast and Menopause Clinic, Ghent University Hospital, C. Heymanslaan 10, B-9000 Gent, Belgium

Supplementary information figure 1: Evaluation form for patient comfort on the standard breastboard.


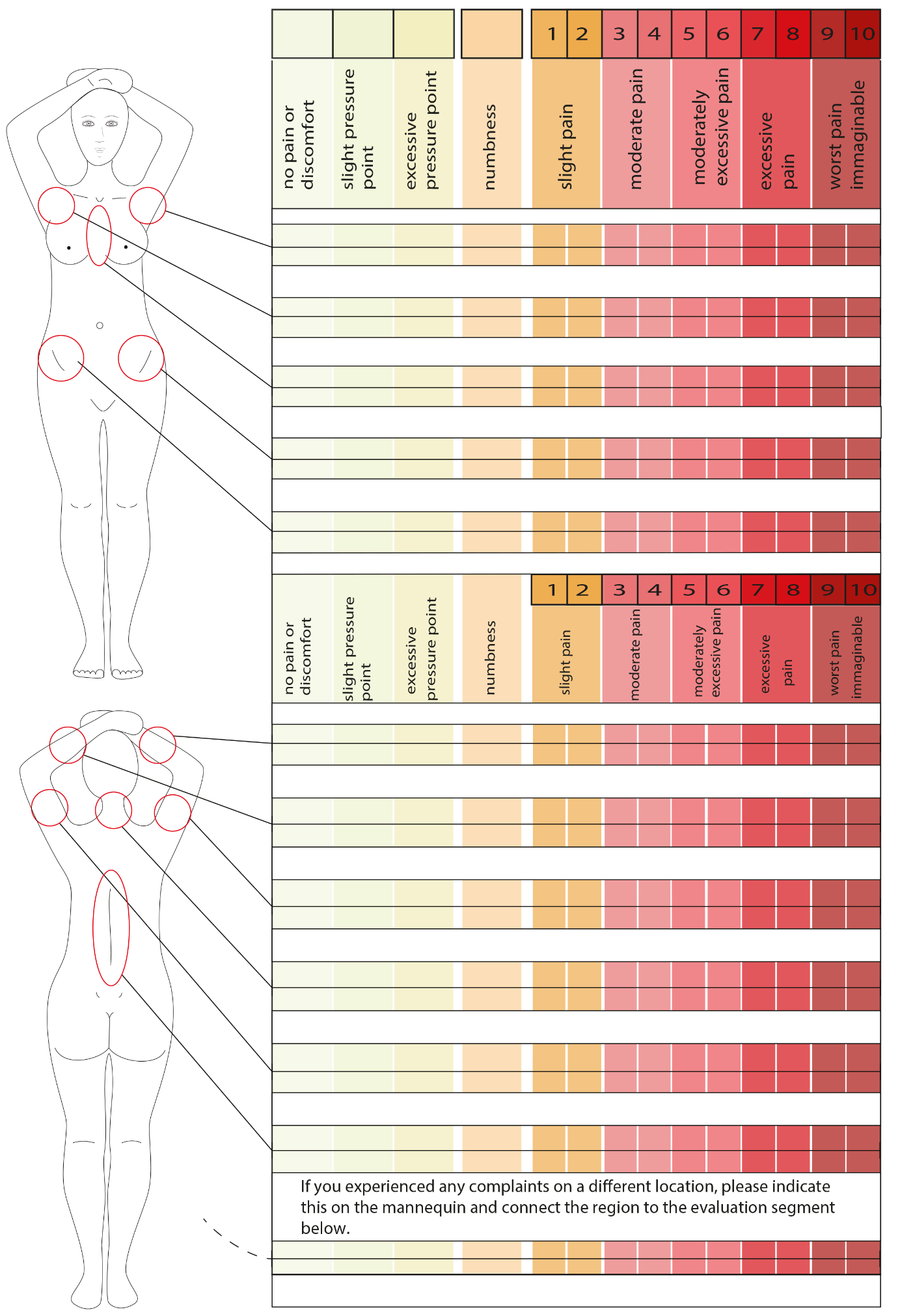


Supplementary information figure 2: Evaluation form for patient comfort on the crawl couch


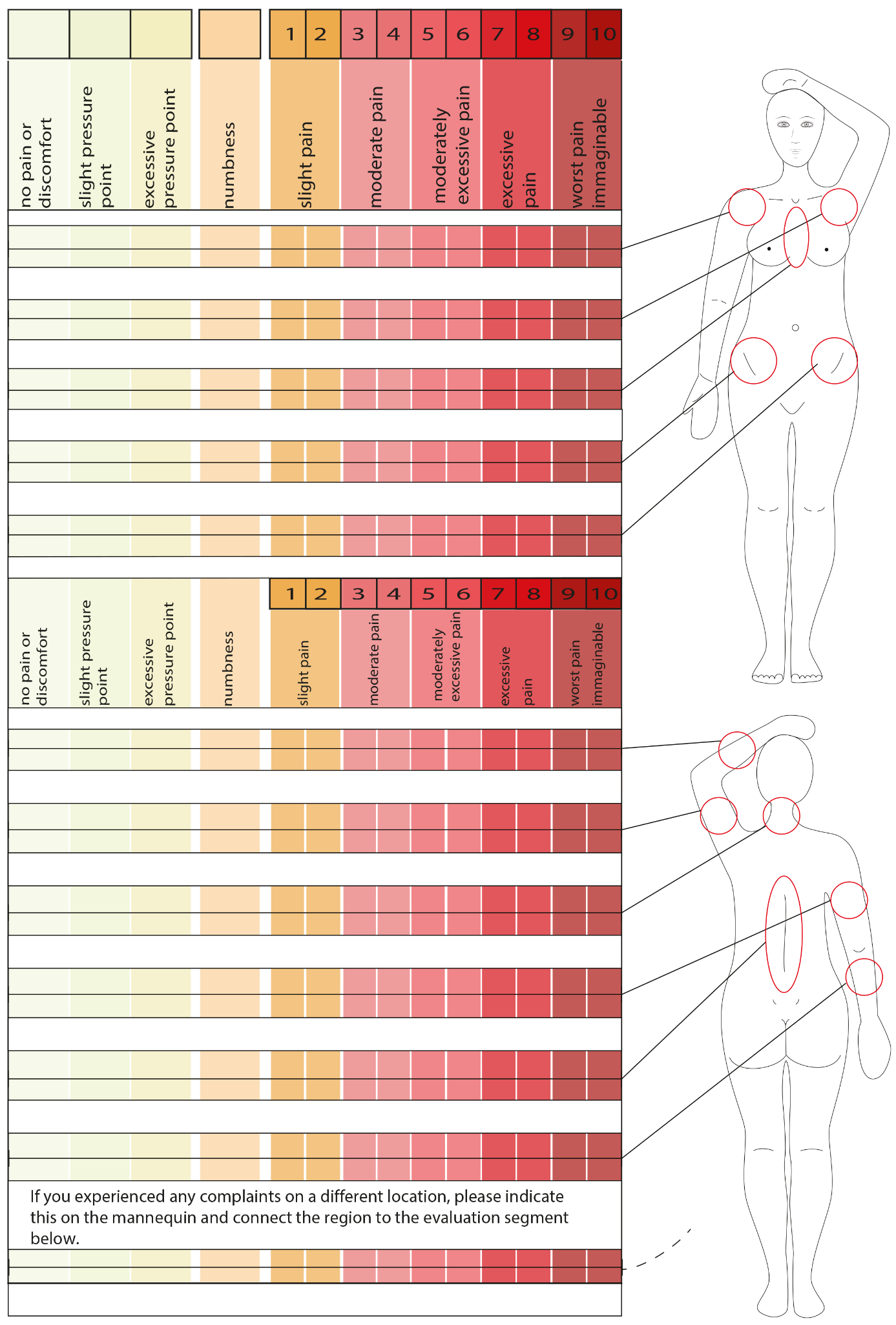

Supplement: Supplementary file 1 — Supplementary Figures. [file 41598_2020_72702_MOESM1_ESM.docx]
